# Effect of Cattaneo-Christov approximation for viscoelastic fluid with carbon nanotubes on flow and heat transfer

**DOI:** 10.1038/s41598-022-13592-5

**Published:** 2022-06-08

**Authors:** U. S. Mahabaleshwar, K. N. Sneha, M. Hatami

**Affiliations:** 1 Department of Mathematics, Davangere University, Shivagangothri, Davangere, 577007 India; 2 Department of Mechanical Engineering, Ferdowsi University of Mashhad, Mashhad, Iran

**Keywords:** Applied mathematics, Other nanotechnology, Mechanical engineering

## Abstract

The current work studies the motion of viscoelastic liquid saturated with carbon nanotubes over a stretching surface in a Darcy porous medium analytically below an influence of Cattaneo-Christov heat flux. The carbon nanotubes (CNTs) act as nanoparticles which are then appended into the base fluid. Water and kerosene are used as a base fluid with two types of CNTs, namely, Single-wall carbon nanotubes and Multiwall carbon nanotubes. Carbon nanotubes possess a wide range of industrial and biomedical applications including energy production, nuclear reactor cooling, and galaxy cooling applications because they can expand the thermal and mechanical properties of base things. As a result, the carbon nanotubes used in the mentioned fields are being investigated for their potential in heat transfer applications. Governing equations formulated using the Partial differential equations have converted to Ordinary differential equations exhausting the appropriate comparison transformation process. An influence of some relevant constraints on velocity and temperature is evaluated in details. The Cattaneo-Christov heat transfer model is utilized to investigate the heat transfer individualities with varying thermal conductivity consuming the attributes of the Appell hypergeometric function. The impacts of the emerging parameters on the profiles are depicted through graphical representations and analytically constructed tables. Considering its usefulness in modulating temperature distribution in different industrial application, including solar collector design, electronic cooling, building ventilation, etc. According to our findings, the temperature profile exhibits an enhancement with the thermal radiation parameter and the viscous-elastic fluids. In addition, when compared to the classical Fourier's law of heat conduction, the temperature profile and thermal boundary layer thickness for the Cattaneo-Christov heat flux model are lower.

## Introduction

A nanofluid flow and heat transfer has become one of the fastest growing areas of nanotechnology engineering and innovation. According to Choi’s survey^1^, there has been drastic growth in researchers publishing. A nanofluid is a combination of base fluid and nanoparticles which is homogeneous. Nanoparticles typically have a diameter of 1–100 nm, but this size can be changed slightly due to shape. Carbon nanotubes (CNTs) were discovered^2–4^ which at room temperature, CNTs have a thermal conductivity that is roughly six times greater than of other materials. CNTs are carbon allotropes having a tube-shaped nanostructure. There are two kinds of CNTs, including SWCNTs and MWCNTs. There are many applications of CNTs in manufacturing and medicine due to their immediate effects for expanding the thermal conductivity of base liquids. These applications include microelectronics cooling, refrigeration, power generation, transportation, air conditioning, chemical processing and others.

From physical point of view, an analysis of boundary layer flows is very useful due to huge applications. It should be noted that boundary layer flow over surfaces differs significantly from free stream flow over stationary plates^5,6^. An impact of magnetic field on boundary layer flow is firstly explored at^7,8^. Several researchers considered the heat and mass transfer using the boundary layer theory under various impacts including non-Newtonian nature of the working fluid, thermal radiation, local heaters, suction/injection, velocity slip, porous media^9–15^. At the same time, some researchers^16–18^ investigated mixed convection boundary layer flow of an incompressible and electrically conducting viscoelastic fluid across a linearly stretching sheet contained porous media.

The energy equation using the original Fourier law was the focus of the prior research. The reason for widespread criticism of the Fourier approach is that it leads to the construction of a parabolic-type energy equation^19^. To get around this constraint, Cattaneo first used a relaxation time term^20^. As an update, Christov^21^ introduced an innovative category derivative of Oldroyd’s upper-convicted variant, and yet this composition changed the organizational structure of the Cattaneo-Christov heat transfer paradigm. Thus, Hayat et al.^22^ have studied an influence of Cattaneo-Christov heat flux on viscoelastic fluid flow due to a linear stretching sheet. They found that boundary layer viscosity is less for Cattaneo-Christov thermal flux approach compared to the Fourier heat conduction law. Cattaneo–Christov double diffusive MHD fluid due to stretching cylinder has been studied by Khan et al.^23^ using the similarity technique. Recent studies on the Cattaneo-Christov heat transfer model can be found in^24–28^. Considering its usefulness in modulating temperature distribution in different industrial application, including solar collector design, electronic cooling, building ventilation, etc.^29–31^. Nadeem et al.^32^ have studied a flow occurs due to linear stretching sheet. For the evaluation of heat flux, Fourier’s law of heat conduction is employed. Yang et al.^33^ Heat transfer and friction drag are carried out for these hybridized ferrites nanoparticles in ferromagnetic Nano-fluids. Utilization of Maxwell-Cattaneo Law for MHD swirling flow through oscillatory disk subject to porous medium studied by the Rauf et al.^34–37^. Various researchers^38,39^ in the past years are collaborating in the nanotechnology field due to their improvement in heat capacity, chemotherapy for cancer, microelectronics, cooling of energy storage devices, cooling of nuclear system, air conditioning, and nanochips, etc. The concept of activation energy and double stratification effects is considered to analyze the flow problem. Thermal relaxation time relaxation time properties are both determined by implementing Cattaneo-Christov heat and mass flux in the energy and mass equation.

The present research is an addition to the prior investigation by Jafarimoghaddam et al.^40^. The present novelty of the work deals with an inclined magnetic field, carbon nanotubes and Darcy porous medium. The Cattaneo-Christov heat flux concept in nanofluid flow is compared using two types of viscoelastic fluids in this paper. Second-grade and elastic-viscous fluid are also discussed. The objective function of ordinary differential equations is demonstrated to have analytical solutions for velocity and temperature equations. The energy method allows obtaining a closed form of analytical expression based on the features of the Appell hypergeometric function of binary variables. Analysis of the single- and multi-walled carbon nanotubes viscoelastic fluid flow over a porous medium with thermal radiation has been performed expending analytical methods. The impacts of various fluid flow parameters, including the Cattaneo-Christov heat flux model on inclined MHD fluid flow, are explained using graphs. Furthermore, the velocity and heat transfer are investigated under various graphs in order to explore their physical implications and to compare the influence of several physical constraints on the velocity and thermal boundaries.

## Physical model and solution

**Figure 1:**
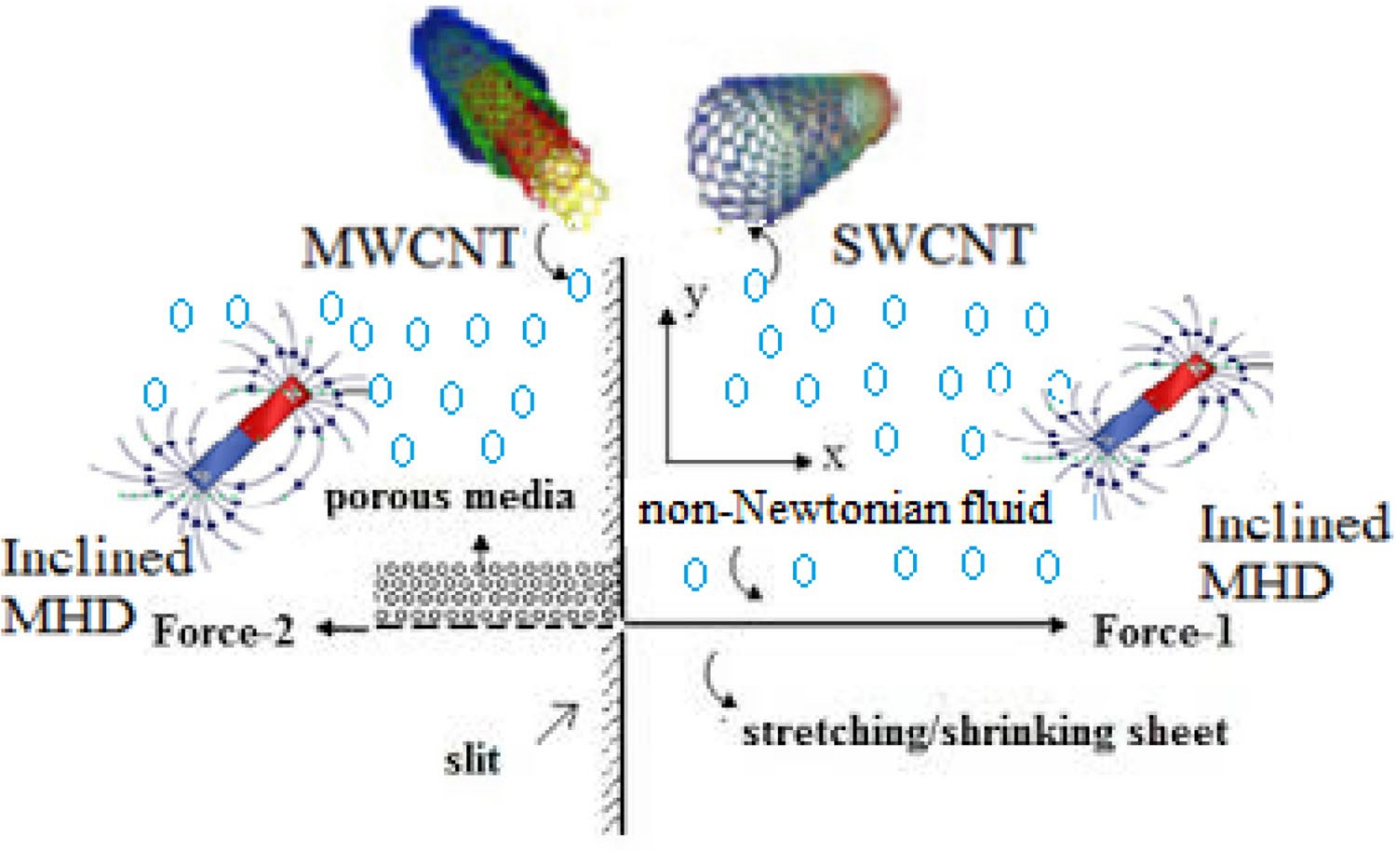
Schematic diagram of stretching boundary.

An investigation of incompressible flow of second grade liquid/Walters’ B liquid created by a continuously stretched sheet under an influence of inclined magnetic field and Darcy porous medium was performed (see Fig. 1). In two instances of fluids, the governing two-dimensional boundary layer flows are examined.

Governing equation can be formulated as follows

1 $$\frac{\partial u}{{\partial x}} + \frac{\partial v}{{\partial y}} = 0$$

2 $$u\frac{\partial u}{{\partial x}} + v\frac{\partial u}{{\partial y}}\, = \,\nu_{nf} \frac{{\partial^{2} u}}{{\partial y^{2} }} - \frac{{\sigma_{nf} B_{0}^{2} }}{{\rho_{nf} }}{\text{sin}}^{2} \left( \tau \right)u - k^{*} \left( {u\,\,\frac{{\partial^{2} u}}{{\partial x\partial y^{2} }} + v\frac{{\partial^{3} u}}{{\partial y^{3} }} - \frac{\partial u}{{\partial y}}\,\frac{{\partial^{2} u}}{\partial x\partial }} \right) - \frac{{\nu_{nf} }}{K}u$$

3 $$\left( {\rho C_{p} } \right)_{nf} \left( {u\frac{\partial T}{{\partial x}} + v\frac{\partial T}{{\partial y}}} \right)\,\,\, = \,\, - \nabla \left( {q + q_{r} } \right)$$

where $k^{*} \, = - {\alpha \mathord{\left/ {\vphantom {\alpha {\rho_{f} }}} \right. \kern-\nulldelimiterspace} {\rho_{f} }}$ is the elastic parameter, k^*^ > 0 is for viscoelastic fluid ^41^, k^*^ < 0 is for the second grade fluid ^42^, while k^*^ = 0 is for the Newtonian fluid ^6^.

Using the Cattaneo-Christov model ^24^ one can find

4 $$q + \lambda \left( {\frac{\partial q}{{\partial t}} + V\,\nabla q - q\nabla V + \left( {\nabla V} \right){\varvec{q}}} \right)\, = - k\nabla T$$

Here *k* is the thermal conductivity and λ is the heat flow relaxation time. Equation (4) can be simplified to the standard Fourier’s equation of heat transfer when λ = 0.

When *q* is included in Eqs. (3) and (4), the temperature profile can be attained as follows

5 $$\begin{gathered} u\frac{\partial T}{{\partial x}} + v\frac{\partial T}{{\partial y}} + \lambda \left( {u\frac{\partial u}{{\partial x}}\frac{\partial T}{{\partial x}} + v\frac{\partial v}{{\partial y}}\frac{\partial T}{{\partial y}} + u\,\frac{\partial v}{{\partial x}}\frac{\partial T}{{\partial y}} + v\frac{\partial u}{{\partial y}}\frac{\partial T}{{\partial x}} + 2uv\frac{{\partial^{2} T}}{\partial x\partial y} + u^{2} \frac{{\partial^{2} T}}{{\partial x^{2} }} + v^{2} \frac{{\partial^{2} T}}{{\partial y^{2} }}} \right) = \hfill \\ = \alpha_{nf} \frac{{\partial^{2} T}}{{\partial y^{2} }} - \frac{1}{{\left( {\rho C_{p} } \right)}}_{nf} \frac{{\partial q_{r} }}{\partial y} \hfill \\ \end{gathered}$$

The following B. Cs are used Jafarimoghaddam et al. ^40^.

6 $$\begin{gathered} u\left( {x,0} \right) = U_{w} = cx,\,\,\,\,V\left( {x,\,0} \right) = 0,\,\,u\left( {x,y \to \infty } \right) = 0, \hfill \\ T\left( {x,0} \right) = T_{w} ,\,\,T\left( {x,y \to \infty } \right) = T_{\infty } . \hfill \\ \end{gathered}$$

Blasius similarity transformation is given by

7 $$u = cxf_{\eta } \left( \eta \right),\,\,v = - \sqrt {c\nu_{f} } f\left( \eta \right), \, \theta \left( \eta \right) = \frac{{T - T_{\infty } }}{{T_{w} - T_{\infty } }},\,\,\,\eta = \sqrt {\frac{c}{{\nu_{f} }}} y$$

Now since Eq. (1) is verified, the method applied results from Eqs. (2) and (5)

8 $$\begin{gathered} \varepsilon_{2} \frac{{d^{3} f}}{{d\eta^{3} }} + \varepsilon_{1} \left( {f\frac{{d^{2} f}}{{d\eta^{2} }} - \left( {\frac{df}{{d\eta }}} \right)^{2} } \right) - \varepsilon_{1} k_{1} \left( {2\left( {\frac{df}{{d\eta }}} \right)\left( {\frac{{d^{3} f}}{{d\eta^{3} }}} \right) - \left( {\frac{{d^{2} f}}{{d\eta^{2} }}} \right)^{2} - f\left( {\frac{{d^{4} f}}{{d\eta^{4} }}} \right)} \right)\, \hfill \\ \,\,\,\,\,\,\,\,\,\,\,\,\,\,\,\,\,\,\,\,\,\,\,\,\,\,\,\,\,\,\,\,\,\,\,\,\,\,\,\,\,\,\,\,\,\,\,\,\,\,\,\,\,\,\,\,\,\,\,\,\,\,\,\,\,\,\,\,\,\,\,\,\,\,\,\,\,\,\,\,\,\,\,\,\, - \left( {\varepsilon_{3} M{\text{sin}}^{2} \left( \tau \right) + \varepsilon_{2} Da^{ - 1} } \right)\left( {\frac{df}{{d\eta }}} \right) = 0 \hfill \\ \end{gathered}$$

9 $$\left( {\varepsilon_{5} + N_{R} } \right)\left( {\frac{{d^{2} \theta }}{{d\eta^{2} }}} \right) + Pr\varepsilon_{4} f\left( {\frac{d\theta }{{d\eta }}} \right)\, - Pr\varepsilon_{4} \gamma \left( {f\left( {\frac{df}{{d\eta }}} \right)\left( {\frac{d\theta }{{d\eta }}} \right) + f^{2} \left( {\frac{{d^{2} \theta }}{{d\eta^{2} }}} \right)} \right) = 0$$

The used B Cs is

10 $$\begin{gathered} f\left( 0 \right) = 0, \, \,\left( {\frac{df}{{d\eta }}} \right)\left( 0 \right) = 1, \, \left( {\frac{df}{{d\eta }}} \right)\left( {\eta \to \infty } \right)\, = 0, \hfill \\ \theta \left( 0 \right) = 1, \, \theta \left( {\eta \to \infty } \right) = 0. \hfill \\ \end{gathered}$$

Here $Da = \frac{Kc}{{\nu_{f} }}$ is the Darcy number,

$k_{1} = - \frac{{ck^{*} }}{{\nu_{f} }}$ is the viscoelastic parameter,

$M = \frac{{\sigma_{f} B_{0}^{2} }}{{\rho_{f} c}}$ is the magnetic parameter,

$Pr = \frac{{\nu_{f} }}{{\alpha_{f} }}$ is the Prandtl number,

$\gamma = c\lambda$ is the relaxation time parameter,

$\alpha_{f} = \frac{{\kappa_{f} }}{{\left( {\rho C_{p} } \right)_{f} }}$ is the thermal diffusivity,

and

$$\varepsilon_{1} = \frac{{\rho_{nf} }}{{\rho_{f} }}, \, \varepsilon_{2} = \frac{{\mu_{nf} }}{{\mu_{f} }}, \, \varepsilon_{3} = \frac{{\sigma_{nf} }}{{\sigma_{f} }}, \, \varepsilon_{4} = \frac{{\left( {\rho C_{p} } \right)_{nf} }}{{\left( {\rho C_{p} } \right)_{f} }}, \, \varepsilon_{5} = \frac{{\kappa_{nf} }}{{\kappa_{f} }}$$

The nanofluids constants are mathematically defined as: (see Muhammad et al.^43–47^)

10a $$\left. \begin{gathered} \nu_{nf} = \frac{{\mu_{nf} }}{{\rho_{nf} }},\;\mu_{nf} = \frac{{\mu_{f} }}{{\left( {1 - \varphi } \right)^{2.5} }}, \hfill \\ \rho_{nf} = \rho_{f} \left( {1 - \varphi } \right) + \varphi \rho_{CNT} \hfill \\ \frac{{\kappa_{nf} }}{{\kappa_{f} }} = \frac{{1 - \varphi + 2\varphi \left( {\frac{{\kappa_{CNT} }}{{\kappa_{CNT} - \kappa_{f} }}} \right)\ln \left( {\frac{{\kappa_{CNT} - \kappa_{f} }}{{2\kappa_{f} }}} \right)}}{{1 - \varphi + 2\varphi \left( {\frac{{\kappa_{f} }}{{\kappa_{CNT} - \kappa_{f} }}} \right)\ln \left( {\frac{{\kappa_{CNT} - \kappa_{f} }}{{2\kappa_{f} }}} \right)}}, \hfill \\ \left( {\rho C_{p} } \right)_{nf} = \left( {1 - \varphi } \right)\left( {\rho C_{p} } \right)_{f} + \varphi \left( {\rho C_{p} } \right)_{CNT} \hfill \\ \end{gathered} \right\}$$

Tables 1 and 2 show the thermal properties of different nanoparticles and base fluids.

**Table 1:** Thermal properties of nanofluid.

Nano liquid physical properties	Liquid phase (water)	Copper	Alumina	Titania
*C*_*p*_(J/kgK)	4179	385	765	686.2
$\rho$(kg/m^3^)	997.1	8933	3970	42.50
*k*(W/mK)	0.613	400	40	8.9538

**Table 2:** Thermophysical properties of water and CNT.

Thermophysical properties	Base liquids				Nanoparticle	
	Water (*Pr* = 6.2)	Ethylene glycol	Engine oil	Kerosene (*Pr* = 21)	SWCNTs	MWCNTs
*Cp*(J/kgK)	997	1.115	884		2.600	1.600
$\rho$(kg/m3)	4.179	2.430	1.910		425	796
*k*(W/mK)	0.613	0.253	0.144		6.600	3.000

The necessary solution is expected to be around the form, based on the exact analytical model for Eq. (8) and the accompanying boundary condition (10) and the preceding choice of *f*_η_ is

11 $$f\left( \eta \right) = \,\frac{{1 - {\text{Exp}}\left( { - \alpha \eta } \right)}}{\alpha },$$

The following equation, which is produced by utilizing Eq. (11) in Eq. (8), allows to determine the unknown

12 $$\alpha \, = \,\sqrt {\frac{{\varepsilon_{1} + \left( {\varepsilon_{3} M{\text{sin}}^{2} \left( \tau \right) + \varepsilon_{2} Da^{ - 1} } \right)}}{{\left( {\varepsilon_{2} - \varepsilon_{1} k_{1} } \right)}}}$$

## Analytical solution for heat transfer

### Analytical solution

Equation (9) is rewritten in the following way

13 $$\theta_{\eta \eta } \left( \eta \right) + h\left( f \right)\theta_{\eta } \left( \eta \right) = 0$$

Using the form of $f_{\eta } \left( \eta \right)\, = 1 - \alpha f\left( \eta \right)$ one can find

14 $$h\left( f \right) = \frac{{\varepsilon_{4} }}{{\left( {\varepsilon_{5} + N_{R} } \right)}}\left[ {\frac{{\left( {\alpha Pr\gamma } \right)f^{2} + Pr\left( {1 - \gamma } \right)f}}{{1 - \gamma Prf^{2} }}} \right]$$

According to the provided boundary conditions, the result for Eq. (13) is expressed as follows:

15 $$\theta \left( \eta \right) = 1 + \theta_{\eta } \left( 0 \right)\int\limits_{0}^{\eta } {Exp\,\left[ { - \int\limits_{0}^{\eta } {\,h\left( f \right)\,d\eta } } \right]} d\eta$$

Equation (15) as $\eta \to \infty$ gives

16 $$\theta_{\eta } \left( 0 \right) = - \frac{1}{{\int\limits_{0}^{\infty } {Exp\,\left[ { - \int\limits_{0}^{\eta } {\,h\left( f \right)\,d\eta } } \right]} d\eta }}$$

17 $$\begin{gathered} - \int\limits_{0}^{\eta } {h\left( f \right)d\eta = - \int\limits_{0}^{f\left( \eta \right)} {\frac{h\left( f \right)}{{\left( {1 - \alpha f\left( \eta \right)} \right)}}} } df\left( \eta \right) = \hfill \\ = ln\left( {\frac{{\left( {1 - \alpha f\left( \eta \right)} \right)^{{ - \left( {\frac{{\varepsilon_{4Pr} }}{{\gamma \,Pr\varepsilon_{4} - \alpha^{2} \left( {\varepsilon_{5} + N_{R} } \right)}}} \right)}} }}{{\left( {1 - sf\left( \eta \right)} \right)^{{ - \left( {\frac{{\alpha \sqrt {Pr\frac{{\varepsilon_{4} }}{{\left( {\varepsilon_{5} + N_{R} } \right)}}} + \sqrt \gamma \left( {Pr\frac{{\varepsilon_{4} }}{{\left( {\varepsilon_{5} + N_{R} } \right)}} + \alpha^{2} - \frac{{\gamma \,Pr\varepsilon_{4} }}{{\left( {\varepsilon_{5} + N_{R} } \right)}}} \right)}}{{2\sqrt \gamma \left( {\frac{{\varepsilon_{4} \,\gamma Pr}}{{\left( {\varepsilon_{5} + N_{R} } \right)}} - \alpha^{2} } \right)}}} \right)}} \left( {1 + sf\left( \eta \right)} \right)^{{ - \left( {\frac{{\alpha \sqrt {Pr\frac{{\varepsilon_{4} }}{{\left( {\varepsilon_{5} + N_{R} } \right)}}} + \sqrt \gamma \left( {Pr\frac{{\varepsilon_{4} }}{{\left( {\varepsilon_{5} + N_{R} } \right)}} + \alpha^{2} - \frac{{\gamma \,Pr\varepsilon_{4} }}{{\left( {\varepsilon_{5} + N_{R} } \right)}}} \right)}}{{2\sqrt \gamma \left( {\frac{{\varepsilon_{4} \,\gamma Pr}}{{\left( {\varepsilon_{5} + N_{R} } \right)}} - \alpha^{2} } \right)}}} \right)}} }}} \right) \hfill \\ \end{gathered}$$

Here we write it in the form

18 $$\begin{gathered} \int\limits_{0}^{\eta } {Exp\,\left[ { - \int\limits_{0}^{\eta } {h\left( f \right)\,d\eta } } \right]d\eta } \, = \hfill \\ = \, - \int\limits_{0}^{f\left( \eta \right)} {\frac{{\left( {1 - \alpha f\left( \eta \right)} \right)^{{ - \left( {\frac{{\varepsilon_{4Pr} }}{{\gamma \,Pr\varepsilon_{4} - \alpha^{2} \left( {\varepsilon_{5} + N_{R} } \right)}}} \right) - 1}} }}{{\left( {1 - sf\left( \eta \right)} \right)^{{ - \frac{{\alpha \sqrt {Pr\frac{{\varepsilon_{4} }}{{\left( {\varepsilon_{5} + N_{R} } \right)}}} + \sqrt \gamma \left( {Pr\frac{{\varepsilon_{4} }}{{\left( {\varepsilon_{5} + N_{R} } \right)}} + \alpha^{2} - \frac{{\gamma \,Pr\varepsilon_{4} }}{{\left( {\varepsilon_{5} + N_{R} } \right)}}} \right)}}{{2\sqrt \gamma \left( {\frac{{\varepsilon_{4} \,\gamma Pr}}{{\left( {\varepsilon_{5} + N_{R} } \right)}} - \alpha^{2} } \right)}}}} \left( {1 + sf\left( \eta \right)} \right)^{{ - \frac{{\alpha \sqrt {Pr\frac{{\varepsilon_{4} }}{{\left( {\varepsilon_{5} + N_{R} } \right)}}} + \sqrt \gamma \left( {Pr\frac{{\varepsilon_{4} }}{{\left( {\varepsilon_{5} + N_{R} } \right)}} + \alpha^{2} - \frac{{\gamma \,Pr\varepsilon_{4} }}{{\left( {\varepsilon_{5} + N_{R} } \right)}}} \right)}}{{2\sqrt \gamma \left( {\frac{{\varepsilon_{4} \,\gamma Pr}}{{\left( {\varepsilon_{5} + N_{R} } \right)}} - \alpha^{2} } \right)}}}} }}} \,df\left( \eta \right) \hfill \\ \end{gathered}$$

Expending the Appell hypergeometric function of two variables one can find

19 $$\begin{gathered} \int\limits_{0}^{f\left( \eta \right)} {\frac{{\left( {1 - \alpha f\left( \eta \right)} \right)^{ - A - 1} }}{{\left( {1 - sf\left( \eta \right)} \right)^{ - B} \left( {1 + sf\left( \eta \right)} \right)^{ - C} }}} \,df\left( \eta \right) = \hfill \\ = \frac{1}{A\alpha }\left( {1 - \alpha f\left( \eta \right)} \right)^{ - A} \,\left( {1 - sf\left( \eta \right)} \right)^{B} \,\left( {1 + sf\left( \eta \right)} \right)^{C} \left( {\frac{\alpha - \alpha sf\left( \eta \right)}{{\alpha - s}}} \right)^{ - B} \times \hfill \\ \times \,\left( {\frac{\alpha + \alpha sf\left( \eta \right)}{{\alpha + s}}} \right)^{ - C} \left. {F_{1} \left( { - A;\, - B,\, - C; - A + 1;\,\frac{{s\left( {\alpha f\left( \eta \right) - 1} \right)}}{\alpha - s},\frac{{ - s\left( {\alpha f\left( \eta \right) - 1} \right)}}{\alpha + s}\,} \right)} \right|_{0}^{f\left( \eta \right)} \hfill \\ \end{gathered}$$

where

20 $$\begin{gathered} A = \,\,\frac{{\varepsilon_{4} }}{{\left( {\varepsilon_{5} + N_{R} } \right)}}\frac{Pr}{{\frac{{\gamma \,Pr\varepsilon_{4} }}{{\left( {\varepsilon_{5} + N_{R} } \right)}} - \alpha^{2} }}, \, B = \frac{{\alpha \sqrt {Pr\frac{{\varepsilon_{4} }}{{\left( {\varepsilon_{5} + N_{R} } \right)}}} + \sqrt \gamma \left( {Pr\frac{{\varepsilon_{4} }}{{\left( {\varepsilon_{5} + N_{R} } \right)}} + \alpha^{2} - \frac{{\gamma \,Pr\varepsilon_{4} }}{{\left( {\varepsilon_{5} + N_{R} } \right)}}} \right)}}{{2\sqrt \gamma \left( {\frac{{\varepsilon_{4} \,\gamma Pr}}{{\left( {\varepsilon_{5} + N_{R} } \right)}} - \alpha^{2} } \right)}}, \hfill \\ C = \frac{{ - \alpha \sqrt {Pr\frac{{\varepsilon_{4} }}{{\left( {\varepsilon_{5} + N_{R} } \right)}}} + \sqrt \gamma \left( {Pr\frac{{\varepsilon_{4} }}{{\left( {\varepsilon_{5} + N_{R} } \right)}} + \alpha^{2} - \frac{{\gamma \,Pr\varepsilon_{4} }}{{\left( {\varepsilon_{5} + N_{R} } \right)}}} \right)}}{{2\sqrt \gamma \left( {\frac{{\varepsilon_{4} \,\gamma Pr}}{{\left( {\varepsilon_{5} + N_{R} } \right)}} - \alpha^{2} } \right)}}, \, s = \sqrt {\frac{{\gamma \,Pr\,\varepsilon_{4} }}{{\left( {\varepsilon_{5} + N_{R} } \right)}}} . \hfill \\ \end{gathered}$$

As a result, the outcome is as follows

21 $$\int\limits_{0}^{\infty } {Exp\left[ { - \int\limits_{0}^{\eta } {h\left( f \right)d\eta } } \right]d\eta } \, = \,\frac{{\left( {\frac{\alpha - s}{\alpha }} \right)^{B} \left( {\frac{\alpha - s}{\alpha }} \right)^{C} }}{{AC\left( {\frac{\alpha + \alpha sf\left( \eta \right)}{{\left( {1 - \alpha f\left( \eta \right)} \right)\left( {\alpha + s} \right)}}} \right)^{ - A} }}\,\,\left. {{}_{2}F_{1} \left( { - A, - B; - B - C;\frac{{ - 2s\alpha + 2\alpha^{2} sf\left( \eta \right)}}{{\left( {\alpha + \alpha sf\left( \eta \right)} \right)\left( {\alpha - s} \right)}}} \right)} \right|_{0}^{{f\left( {\eta \to \infty } \right) = \frac{1}{\alpha }}}$$

After applying the integration one can find

22 $$\mathop {\lim }\limits_{\eta \to 0;\,f\left( \eta \right) \to 0} \left( {\int {e^{{ - \int\limits_{0}^{\eta } {h\left( f \right)d\eta } }} \,d\eta } } \right)\, = \,\frac{{\left( {\frac{\alpha - s}{\alpha }} \right)^{B} \left( {\frac{\alpha - s}{\alpha }} \right)^{C - A} }}{AC}\,\,\,{}_{2}F_{1} \left( { - A, - B; - B - C;\frac{ - 2s}{{\left( {\alpha - s} \right)}}} \right).$$

For this case here $f\left( \eta \right) \to \frac{1}{\alpha }$

23 $$\mathop {\lim }\limits_{{\eta \to 0;\,f\left( \eta \right) \to \frac{1}{\alpha }}} \left( {\int {e^{{ - \int\limits_{0}^{\eta } {h\left( f \right)d\eta } }} d\eta } } \right)\, = \,\left\{ \begin{gathered} \to \infty \,A\, > \,0 \hfill \\ \to \,0\,\,A < 0\, \hfill \\ \end{gathered} \right.$$

It is clear that in the case of *A* > 0, the thermal problem solution deviates from its correct physical meaning; as a result *A* < 0 remains to solve the possible solution. Therefore,

24 $$A < \,0,\,\,f_{\eta \eta } (0)^{2} > \frac{{\gamma Pr\varepsilon_{4} }}{{\left( {\varepsilon_{5} + N_{R} } \right)}}$$

The recently discovered threshold condition for the presence of a thermal solution become

25 $$\gamma \left( {\varepsilon_{2} - \varepsilon_{1} k_{1} } \right) < \,\left[ {\varepsilon_{1} + \left( {\varepsilon_{3} M{\text{sin}}^{2} \left( \tau \right) + \varepsilon_{2} Da^{ - 1} } \right)} \right]$$

If the circumstances $f_{\eta \eta } \left( 0 \right)^{2} \, > \gamma \,$ is available

26 $$\theta_{\eta } \left( 0 \right)\,\, = \,\frac{{A\alpha \left( {\frac{\alpha }{\alpha - s}} \right)^{B} \left( {\frac{\alpha }{\alpha - s}} \right)^{C - A} }}{{{}_{2}F_{1} \left( { - A, - B; - B - C;\frac{ - 2s}{{\left( {\alpha - s} \right)}}} \right)}}$$

27 $$\theta \left( \eta \right)\,\, = \,\,\,\,\,\,\,\left( {\frac{{1 + \frac{s}{\alpha }\left( {1 - Exp\left( { - \alpha \eta } \right)} \right)}}{{Exp\left( { - \alpha \eta } \right)}}} \right)^{A} \frac{{{}_{2}F_{1} \left( { - A, - B; - B - C;\frac{{ - 2s\,\alpha Exp\left( { - \alpha \eta } \right)}}{{\left( {\alpha - s} \right)\left[ {s\left( {1 - Exp\left( { - \alpha \eta } \right)} \right) + \alpha } \right]}}} \right)}}{{{}_{2}F_{1} \left( { - A, - B; - B - C;\frac{ - 2s}{{\left( {\alpha - s} \right)}}} \right)}}$$

### Validation study

Here, we note that,

(i) The Crane 1970 flow is recovered from Eq. (12) for $Q = 0\,\,,\,\,k_{1} = \,0,\,Da^{ - 1} \, = \,0,\,\varepsilon_{1} = \varepsilon_{2} = \varepsilon_{3} = 1$.
(ii) The Pavlov 1970 flow is recovered from Eq. (12) for $M = 1\,\,,\,\,k_{1} = \,0,\,Da^{ - 1} \, = \,0,\varepsilon_{1} = \varepsilon_{2} = \varepsilon_{3} = 1$ and $\tau = 90^{0}$ .
(iii) The Mahabaleshwar et al. (2014) flow is recovered from Eq. (12) for $M = 1\,,\,\,k_{1} = \,1,\,\,Da^{ - 1} \, = \,0,\varepsilon_{1} = \varepsilon_{2} = \varepsilon_{3} = 1$.
(iv) The Mahabaleshwar et al. (2005) flow is recovered from Eq. (12) for $\,\,M = 1\,,\,\,k_{1} = \,1,\,\,Da^{ - 1} \, = \,0,\varepsilon_{1} = \varepsilon_{2} = \varepsilon_{3} = 1$.
(v) The Mahabaleshwar et al. (2018) flow is recovered from Eq. (12) for $M = 1\,\,,\,\,k_{1} = \,1,\,Da^{ - 1} \, = \,0,\varepsilon_{1} = \varepsilon_{2} = \varepsilon_{3} = 0$.
(vi) With higher values of each physical properties, the heat transfer improves.
(vii) When comparative to SWCNTs, the base fluid MWCNTs provides superior heat transmission.
(viii) The thickness of the thermal boundary layer enhance as the radiation number rises.
(ix) Present work $M = 1\,,\,\,k_{1} = \,1,\,\,Da^{ - 1} \, = \,1,\varepsilon_{1} = \varepsilon_{2} = \varepsilon_{3} \ne 0$ and $\tau = 90^{0}$ .

The following Table 3 shows related works by other authors and finding existing results.

**Table 3:** Expression for ${\varvec{\alpha}}$ various physical parameters.

Authors	Fluids	Value of ${\varvec{\alpha}}$
Crane 1970	Newtonian	$\alpha = 1$
Pavlov 1974	Newtonian	$\alpha \, = \,\sqrt {1 + M}$
Mahabaleshwar et al*.* 2014	Non-Newtonian	$\alpha \, = \,\sqrt {\frac{1 + Q + K}{{\left( {1 - k_{1} } \right)}}}$
Siddheshwar and Mahabaleshwar 2005	Non-Newtonian	$\alpha \, = \,\sqrt {\frac{1 + Q}{{\left( {1 - k_{1} } \right)}}}$
Mahabaleshwar et al*.* 2018,	Non-Newtonian	$\lambda \alpha^{3} \, + \left( {1 - Re\,k_{1} } \right)\alpha^{2} - \lambda \alpha - \left( {1 + Re} \right) = 0$
Amin et al*.* 2021	Non-Newtonian	Skin friction $f_{\eta \eta } \left( 0 \right) = \, - \sqrt {\frac{1 + M + K}{{\left( {1 - k_{1} } \right)}}}$
Amin et al*.* 2021	Non-Newtonian	$\theta_{\eta } \left( 0 \right)\,\, = \,\frac{{Ac\left( {\frac{c}{c - s}} \right)^{B} \left( {\frac{c}{c - s}} \right)^{C - A} }}{{{}_{2}F_{1} \left( { - A, - B; - B - C;\frac{ - 2s}{{\left( {c - s} \right)}}} \right)}}$ $A = \,\,\frac{Pr}{{\gamma \,Pr - c^{2} }}$, $B = \frac{{c\sqrt {Pr} + \sqrt \gamma \left( {Pr + c^{2} - \gamma \,Pr} \right)}}{{2\sqrt \gamma \left( {\gamma Pr - c^{2} } \right)}},$ $C = \frac{{ - c\sqrt {Pr} + \sqrt \gamma \left( {Pr + c^{2} - \gamma \,Pr} \right)}}{{2\sqrt \gamma \left( {\gamma Pr - c^{2} } \right)}}$,$s = \sqrt {\gamma \,Pr}$
Amin et al*.* 2021	Non-Newtonian	$$\begin{gathered} \theta \left( \eta \right)\,\, = \,\,\,\,\,\,\,\left( {\frac{{1 + \frac{s}{\alpha }\left( {1 - Exp\left( { - \alpha \eta } \right)} \right)}}{{Exp\left( { - \alpha \eta } \right)}}} \right)^{A} \hfill \\ \frac{{{}_{2}F_{1} \left( { - A, - B; - B - C;\frac{{ - 2s\,\alpha Exp\left( { - \alpha \eta } \right)}}{{\left( {\alpha - s} \right)\left[ {s\left( {1 - Exp\left( { - \alpha \eta } \right)} \right) + \alpha } \right]}}} \right)}}{{{}_{2}F_{1} \left( { - A, - B; - B - C;\frac{ - 2s}{{\left( {\alpha - s} \right)}}} \right)}} \hfill \\ \end{gathered}$$
Present work	Non-Newtonian	$\alpha \, = \,\sqrt {\frac{{\varepsilon_{1} + \left( {\varepsilon_{3} M{\text{sin}}^{2} \left( \tau \right) + \varepsilon_{2} Da^{ - 1} } \right)}}{{\left( {\varepsilon_{2} - \varepsilon_{1} k_{1} } \right)}}}$
Temperature	Non-Newtonian	$$\begin{gathered} \theta \left( \eta \right)\,\, = \,\,\,\,\,\,\,\left( {\frac{{1 + \frac{s}{\alpha }\left( {1 - Exp\left( { - \alpha \eta } \right)} \right)}}{{Exp\left( { - \alpha \eta } \right)}}} \right)^{A} \hfill \\ \frac{{{}_{2}F_{1} \left( { - A, - B; - B - C;\frac{{ - 2s\,\alpha Exp\left( { - \alpha \eta } \right)}}{{\left( {\alpha - s} \right)\left[ {s\left( {1 - Exp\left( { - \alpha \eta } \right)} \right) + \alpha } \right]}}} \right)}}{{{}_{2}F_{1} \left( { - A, - B; - B - C;\frac{ - 2s}{{\left( {\alpha - s} \right)}}} \right)}} \hfill \\ \end{gathered}$$ $A = \,\,\frac{{\varepsilon_{4} }}{{\left( {\varepsilon_{5} + N_{R} } \right)}}\frac{Pr}{{\frac{{\gamma \,Pr\varepsilon_{4} }}{{\left( {\varepsilon_{5} + N_{R} } \right)}} - \alpha^{2} }}$, $B = \frac{{\alpha \sqrt {Pr\frac{{\varepsilon_{4} }}{{\left( {\varepsilon_{5} + N_{R} } \right)}}} + \sqrt \gamma \left( {Pr\frac{{\varepsilon_{4} }}{{\left( {\varepsilon_{5} + N_{R} } \right)}} + \alpha^{2} - \frac{{\gamma \,Pr\varepsilon_{4} }}{{\left( {\varepsilon_{5} + N_{R} } \right)}}} \right)}}{{2\sqrt \gamma \left( {\frac{{\varepsilon_{4} \,\gamma Pr}}{{\left( {\varepsilon_{5} + N_{R} } \right)}} - \alpha^{2} } \right)}},$ $C = \frac{{ - \alpha \sqrt {Pr\frac{{\varepsilon_{4} }}{{\left( {\varepsilon_{5} + N_{R} } \right)}}} + \sqrt \gamma \left( {Pr\frac{{\varepsilon_{4} }}{{\left( {\varepsilon_{5} + N_{R} } \right)}} + \alpha^{2} - \frac{{\gamma \,Pr\varepsilon_{4} }}{{\left( {\varepsilon_{5} + N_{R} } \right)}}} \right)}}{{2\sqrt \gamma \left( {\frac{{\varepsilon_{4} \,\gamma Pr}}{{\left( {\varepsilon_{5} + N_{R} } \right)}} - \alpha^{2} } \right)}}$, $s = \sqrt {\frac{{\gamma \,Pr\,\varepsilon_{4} }}{{\left( {\varepsilon_{5} + N_{R} } \right)}}}$
Nusselt number	Non-Newtonian	$\theta_{\eta } \left( 0 \right)\,\, = \,\frac{{A\alpha \left( {\frac{\alpha }{\alpha - s}} \right)^{B} \left( {\frac{\alpha }{\alpha - s}} \right)^{C - A} }}{{{}_{2}F_{1} \left( { - A, - B; - B - C;\frac{ - 2s}{{\left( {\alpha - s} \right)}}} \right)}}$

## Results and discussion

The investigation is simplified in addition by the scoping review of the velocity and temperature equations, which results in a set of ODEs. Exact analytical solutions for momentum and temperature profiles can be achieved by using the appropriate similarity variable. As a result of the multiple graphs presented above that build on the subject, we now understand the technology involved in such fascinating dynamics. Furthermore, the numbers of similar visuals provide a comparative of the transverse, axial, and temperature profiles of SWCNT and MWCNT with the solid volume fraction fixed, with dashed lines reflecting SWCNTs and solid lines indicating MWCNTs.

**Figure 2:**
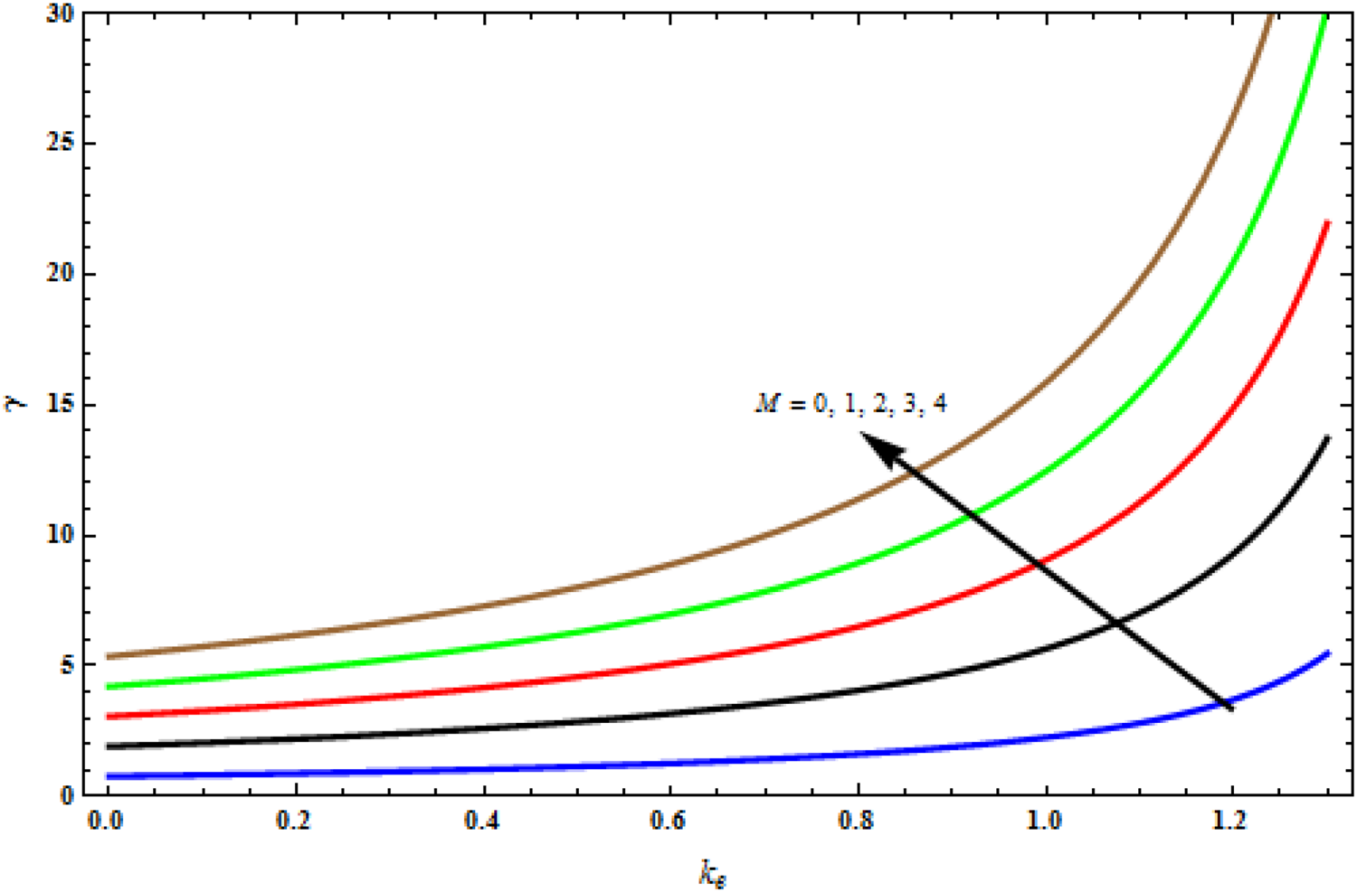
Critical curves illustrating *k*_1_ versus γ with *Pr* = 1, *Da*^-1^ = 1, τ = 90° for stretching case.

Figure 2 demonstrates the relaxation time versus viscoelastic parameter for varying magnetic parameter *M*. The difference is essential because it gives acute curves and limitations for the thermal explanations that will happen after the overall non-Fourier temperature profile is implanted. The magnetic constraint increases the extent of the boundary layer increases in the stretching surface.

**Figure 3:**
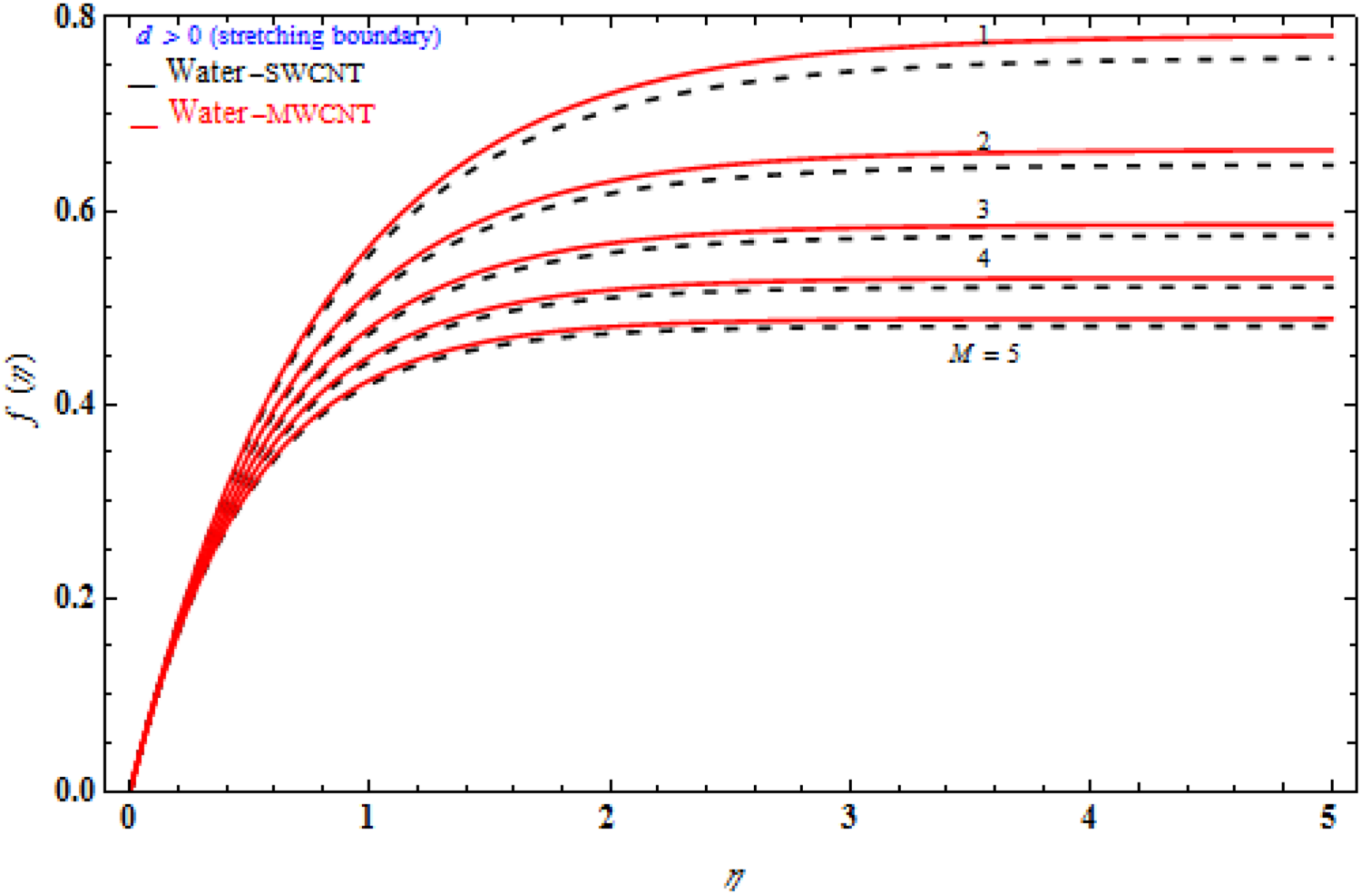
Impact of magnetic field *M* on transverse velocity with *k*_1_ = 1, *Da*^-1^ = 1, τ = 90° for stretching case.

**Figure 4:**
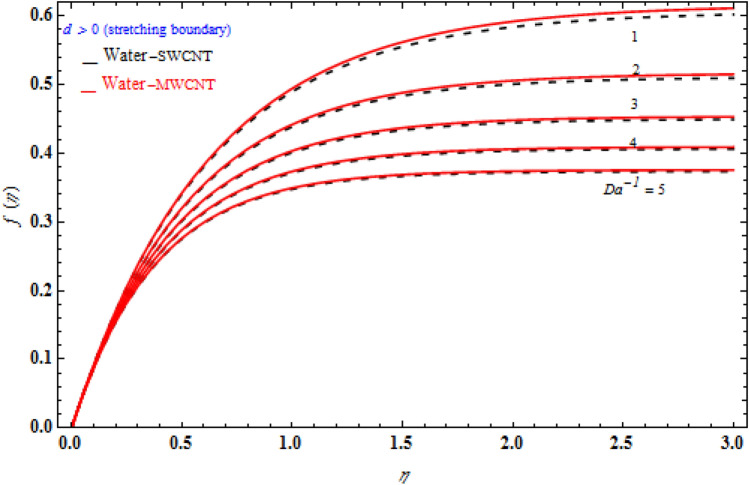
Influence of inverse Da^-1^ on *f* (η) with *k*_1_ = 1, *M* = 1, τ = 90° for stretching case.

Figure 3 signifies the depiction of Darcy model *Da*^-1^ on *f (η)* while the stretching sheet is more than zero. The *f* (η) of MWCNT is more than that of SWCNT. The *f (η)* enhances as the Darcy number also increases. The magnetic field’s inclined parameter is 90 degree. Figure 4 portrays the various values of magnetic parameter *M* on *f* (η) with deference to the similarity variable *η* at the point when the stretching boundary layer is more than nothing. As seen in the diagram, the Lorentz effect is reduced when the raising the $M$ decrease the thickness of the boundary layer, resulting in increased shear stress on the wall. The reason for this is because when the magnetic parameter rise, the boundary layer increases, which is accompanied by a increase in the velocity gradient. In addition, the volume fraction is enhances, the boundary layer thickness enhances.

**Figure 5:**
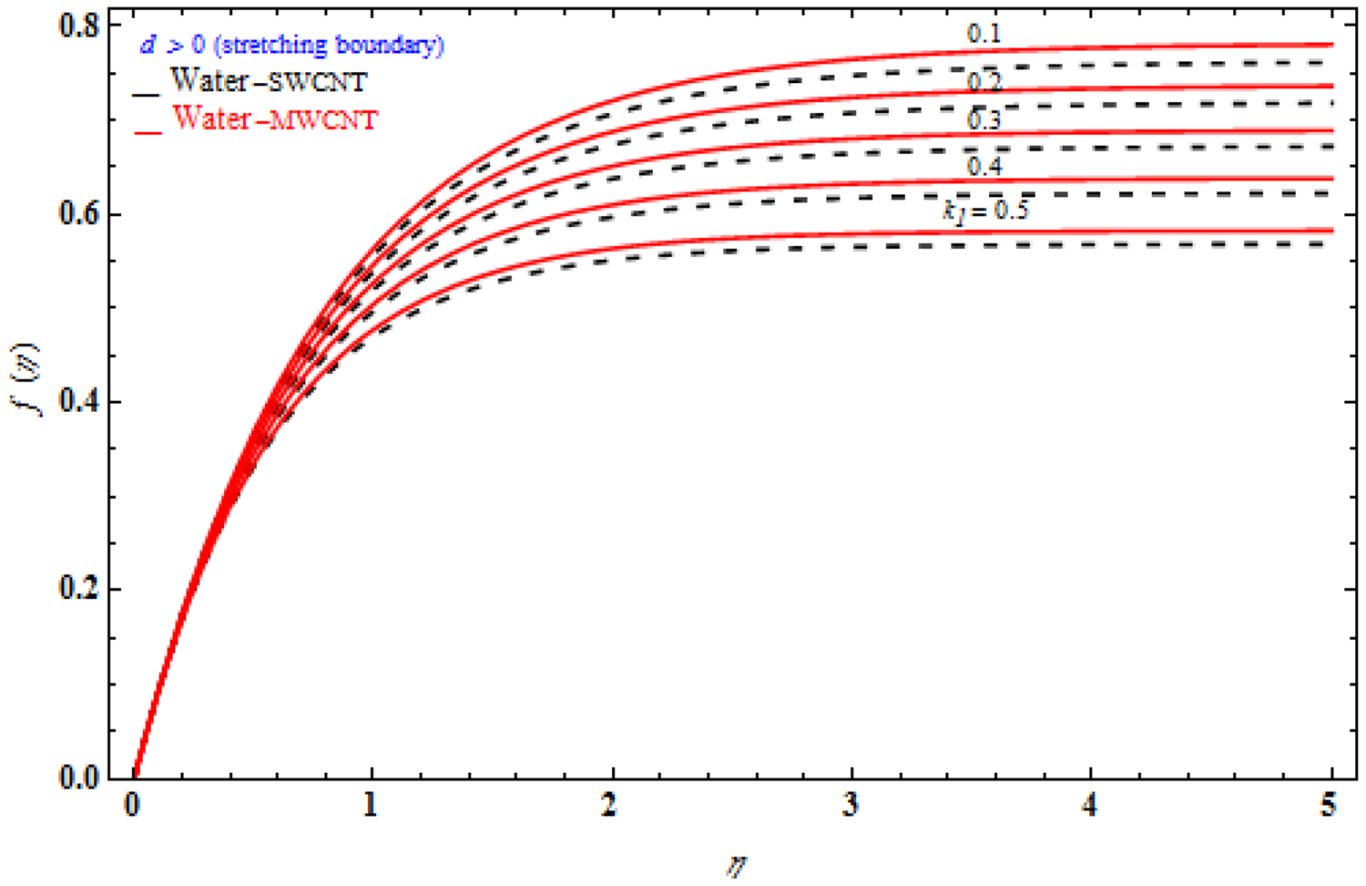
Impact of *k*_1_ on transverse velocity with *M* = 1, *Da*^-1^ = 1, τ = 90° for stretching case.

Figure 5 portrays the various values of viscoelastic *k*_1_ on transverse velocity *f* (η) regarding the similarity variable *η* at the point when the stretching boundary layer is bigger than nothing. The viscoelastic constraint raises the extent of the boundary layer enhances in the stretching surface. The transverse velocity which occurs where there is porous stretching/shrinking sheet. In the presence of stronger viscoelastic fluid higher, will increase transverse velocity that would show off in higher altitudes, namely η ≥ 1. The reason is behind complex rheological behavior of Walter´s liquid B which shows more strength where there is more velocity. At higher altitude where η ≥ 1, the third term in $2f_{\eta } f_{\eta \eta \eta } - ff_{\eta \eta \eta \eta } - f_{\eta \eta }^{2}$ representing shear stress inside fluid layers gradually vanishes and hence cause whole the term to grow. Consequently, it is expected that at higher altitude, effect of viscoelastic fluid would be bolder.

**Figure 6:**
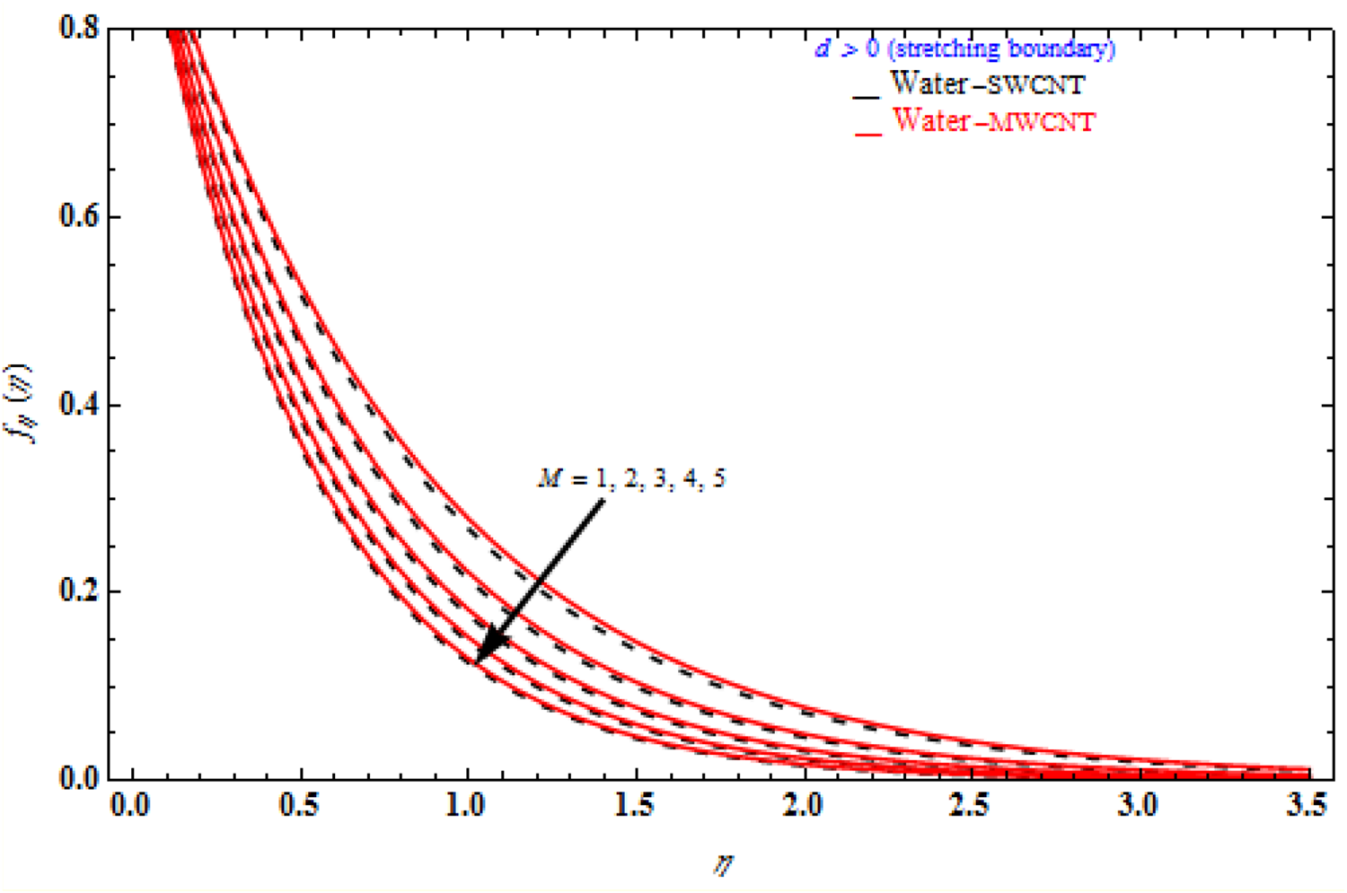
Impact of *M* on axial velocity with *k*_1_ = 1, *Da*^-1^ = 1, τ = 90° for stretching case.

**Figure 7:**
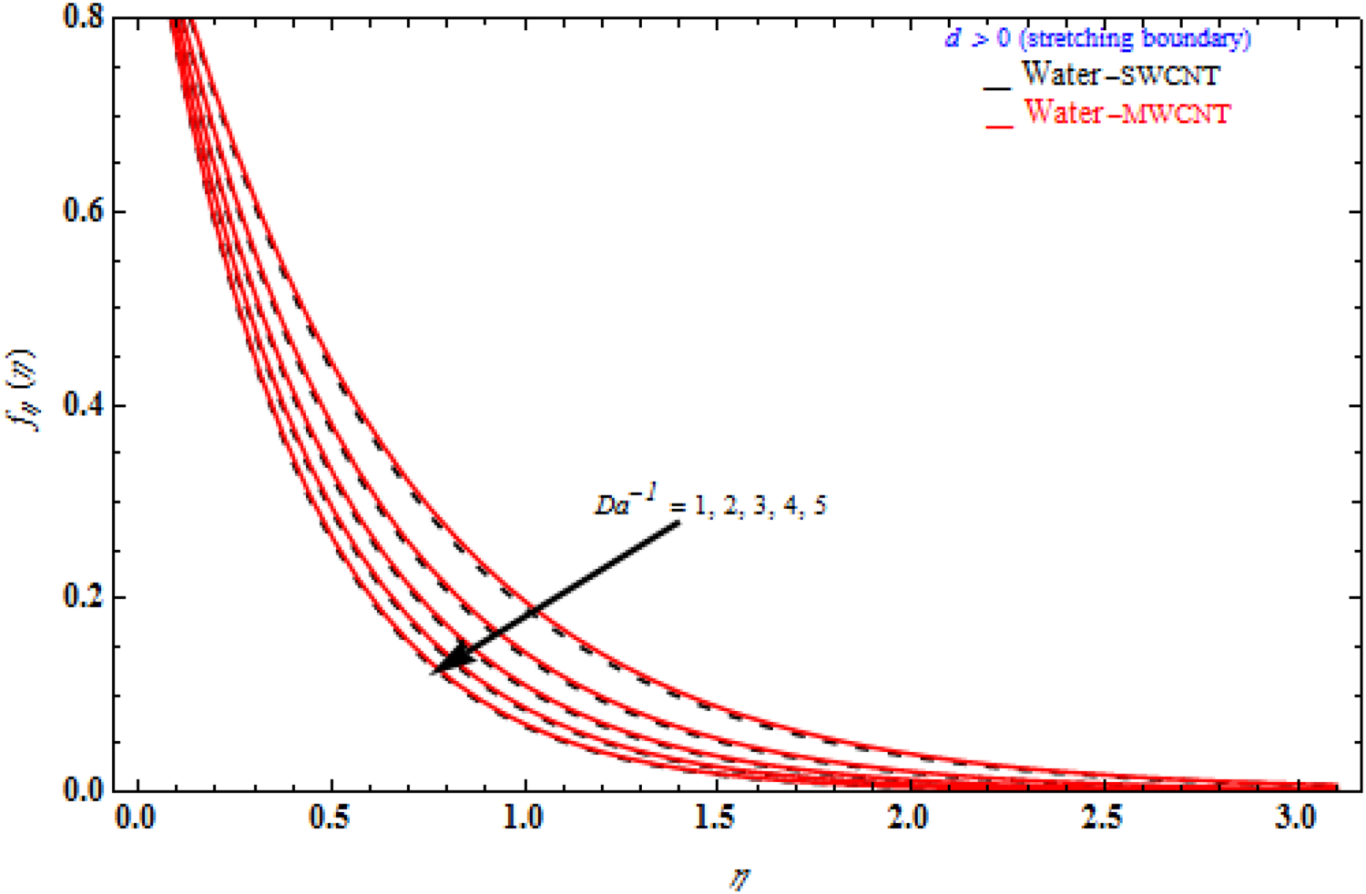
Influence of *Da*^-1^ on axial velocity with *k*_1_ = 1, *M* = 1, τ = 90° for stretching case.

**Figure 8:**
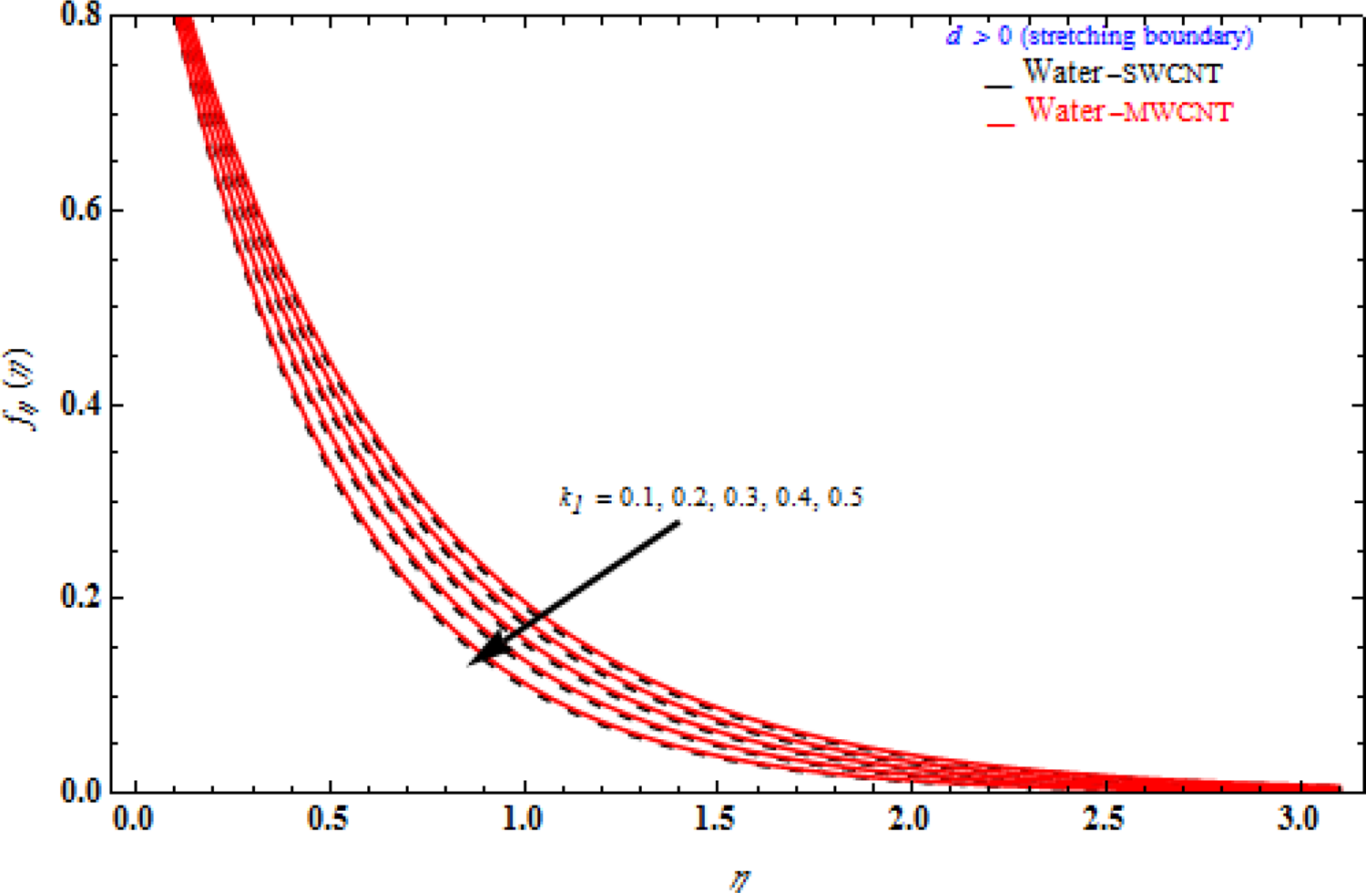
Impact of *k*_1_ on axial velocity with *Da*^-1^ = 1, *M* = 1, τ = 90° for stretching case.

Figures 6, 7 and 8 demonstrate the behaviour of the $f_{\eta } \left( \eta \right)$ on the similarity variable *η* for altered values of Darcy number and magnetic constraint. Figure 6 show that when the magnetic rises, the boundary layer thickness also decreases. In both cases of stretching/shrinking the surface, raising the magnetic parameter increases the axial velocity in the flow field. The influence of magnetic field on velocity at wall for the stretched sheet is seen in this diagram, with velocity and overall axial velocity decreasing as the magnetic field magnitude increases. Magnetic field, as previously stated, is an attractive body force whose projection on the x-axis is in the negative x-direction. It indicates that a larger magnetic field value will cause more axial velocity obstruction and, as a result, will diminish it. The imposed boundary condition and magnetic field, on the other hand, are both in the same direction for shrinking sheet. Consequently, the higher magnetic parameter (as a representative of magnetic field) leads to a higher axial velocity. The similar effect is observed at Fig. 7, it means that $f_{\eta } \left( \eta \right)$ decreases with increasing the Darcy model. The similar effect is observed at Fig. 8, it means that $f_{\eta } \left( \eta \right)$ decreases with increasing the viscoelastic parameter. As can be observed, the viscoelastic influence on axial velocity varies depending on whether the object is shrinking or stretching. In reality, while stretching a sheet, the force and influence of the boundary condition is more important than the fluid's rheology. An increase in the size of the viscoelastic parameter leads in a comparable rise in the axial velocity profiles in both porous stretching and shrinking instances. The boundary layer thins as a result of non-Newtonian viscoelastic shear stress. Furthermore, the boundary layer is smaller when suction is utilized instead of injection.

**Figure 9:**
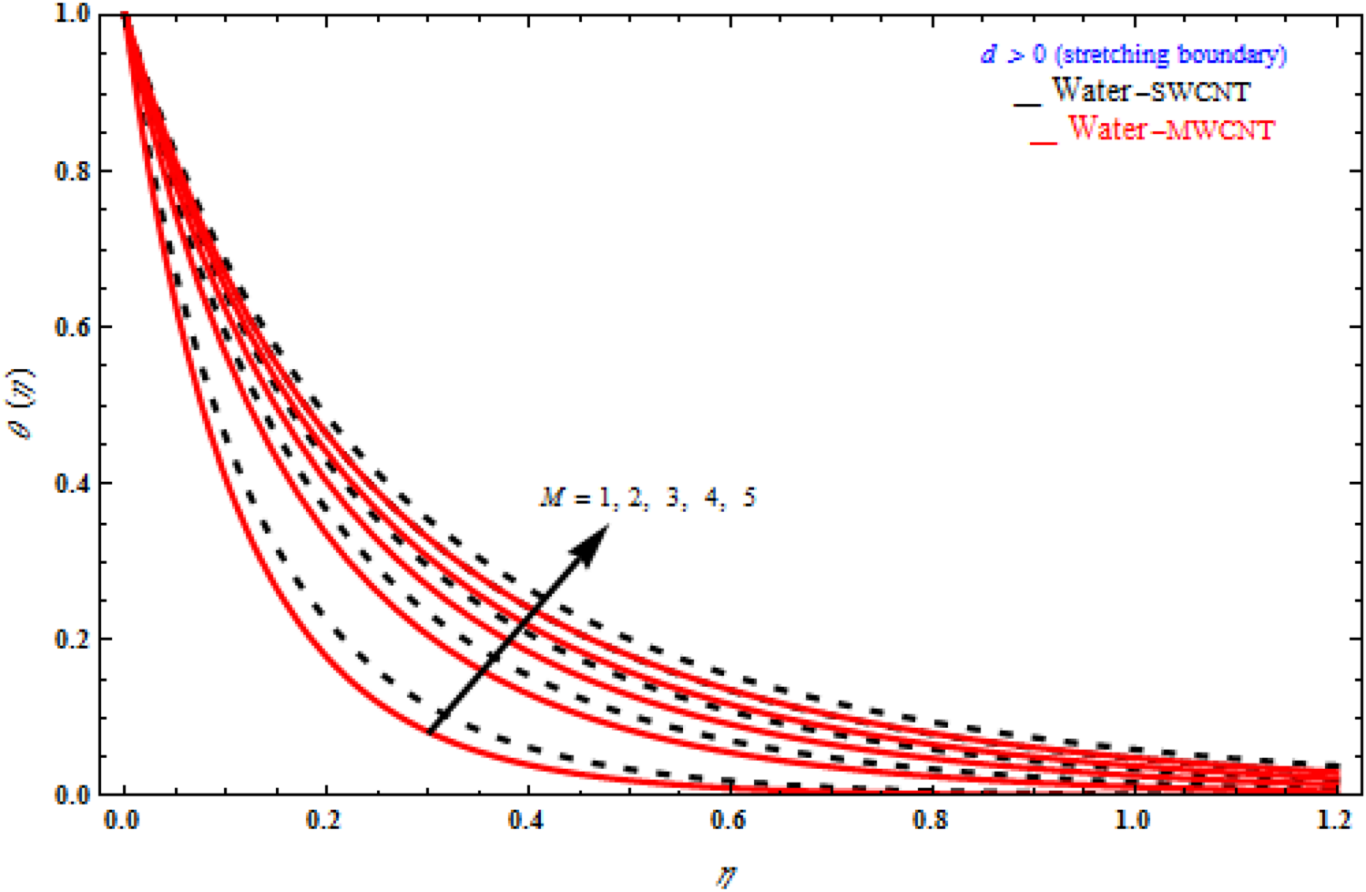
Influence of *M* on temperature with *k*_1_ = 1, *Da*^-1^ = 1, *N*_*R*_ = 1, τ = 90° for stretching case.

**Figure 10:**
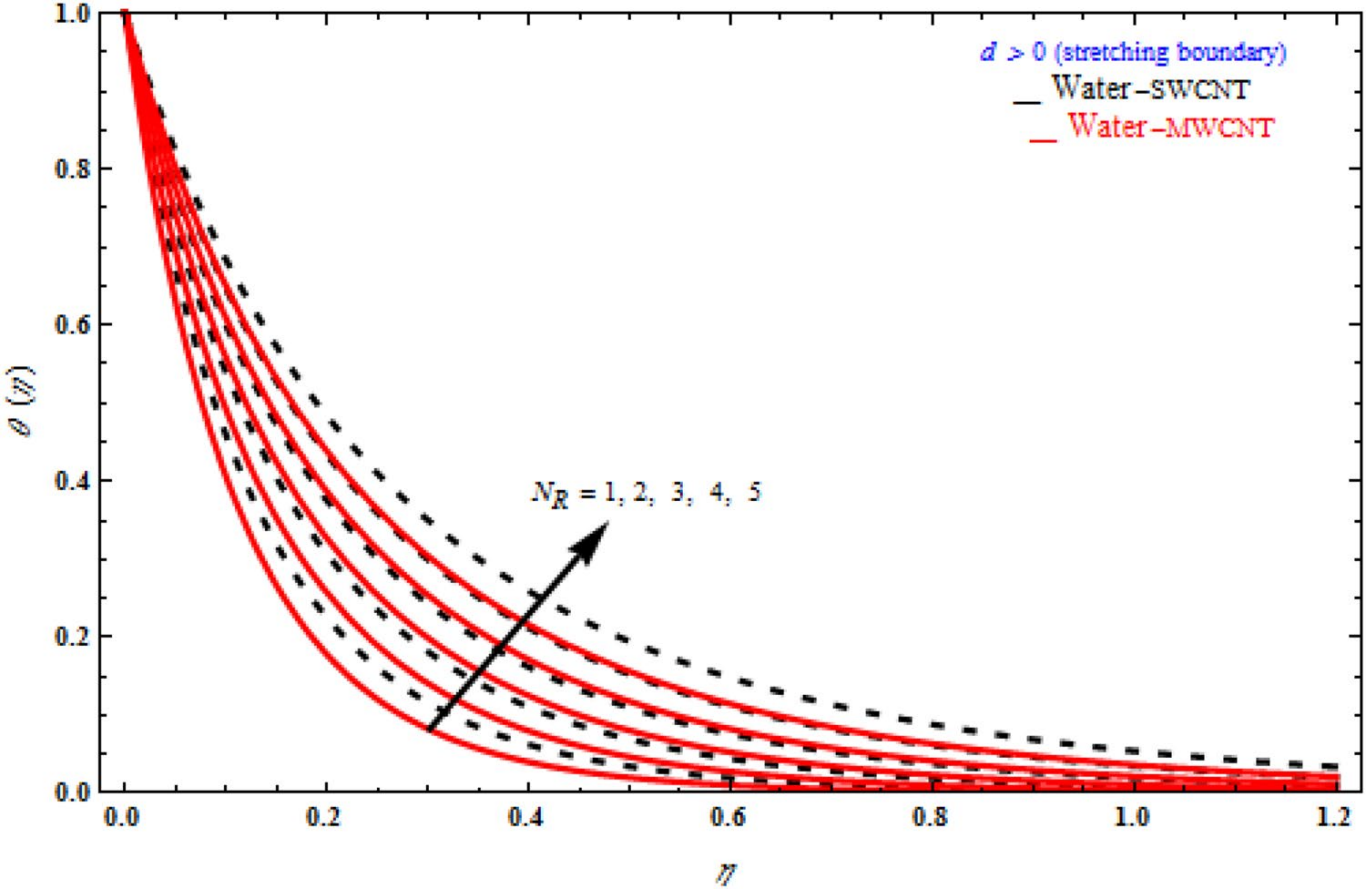
Impact of radiation parameter *N*_*R*_ on temperature profile with *k*_1_ = 1, *Da*^-1^ = 1, *M* = 1, τ = 90° for stretching case.

**Figure 11:**
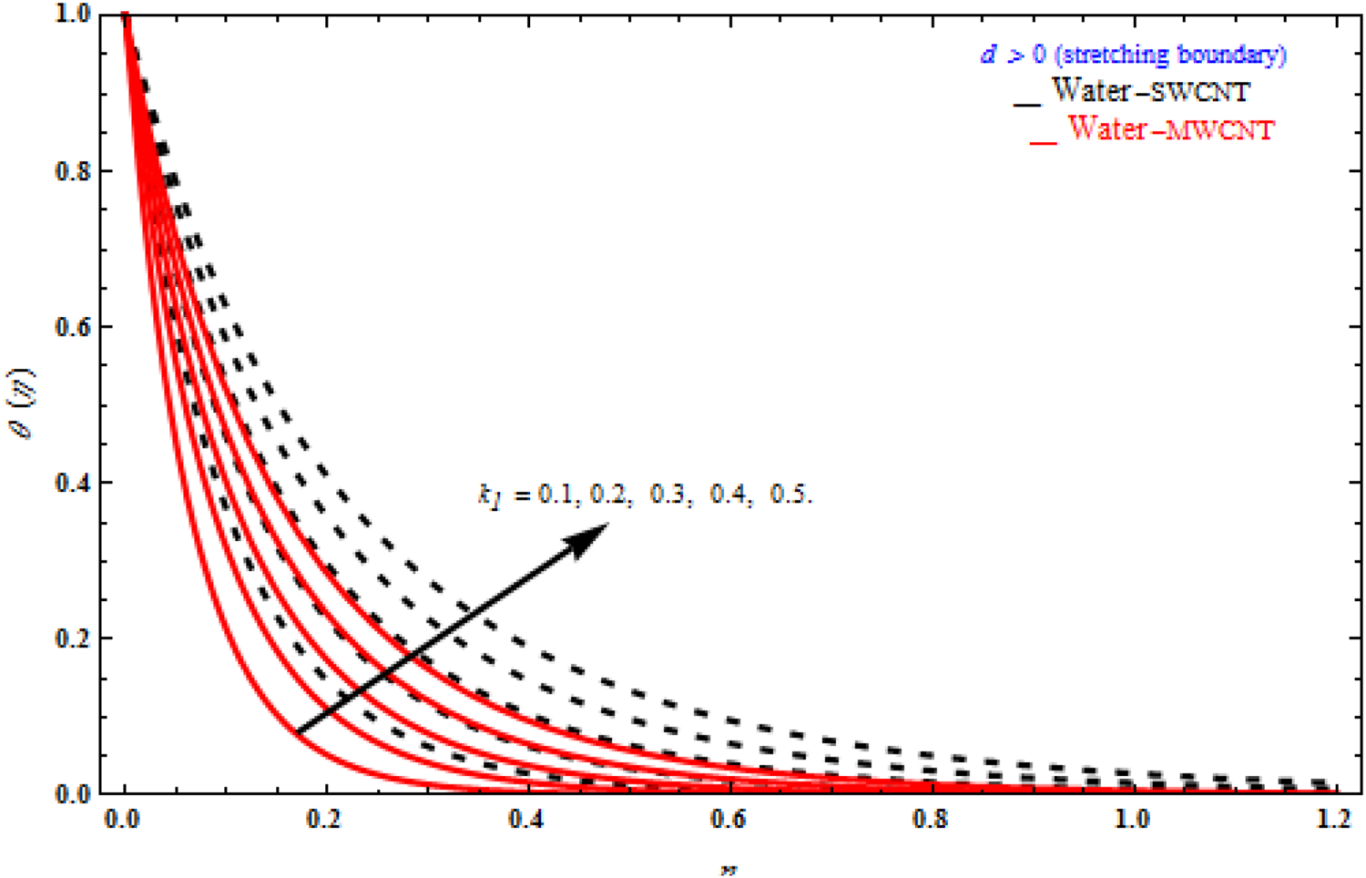
Impact of *k*_1_ on temperature profile with *M* = 1, *Da*^-1^ = 1, *N*_*R*_ = 1, τ = 90° for stretching case.

Figures 9, 10 and 11 represent the temperature fields θ(η) on the similarity variableη, at the point when the stretching limit is more noteworthy than nothing. In Fig. 9, the magnetic parameter increases, the temperature profile also increases. Figure 10 shows different values of radiation. When θ(η) is enhanced, the radiation parameter also rises. Consequently, thermal radiation improves the nanofluids thermal diffusivity, i.e., for emergent values of radiation parameter $N_{R}$ , heat will be supplemented to the regime and temperatures improved accordingly. As mentioned for heat transfer of flows over a stretching sheet, fluid temperature higher than both the wall temperature and the ambient temperature near the wall is physically achievable. Here we discuss of forced flow over a stretching sheet, we now look at heat transport in the presence of radiation. The effect of heat conductivity is amplified by the radiation. Radiation has the effect of dampening or enhancing heat transmission in a linear manner. In Fig. 11, the viscoelastic parameter increases, the temperature profile also increases. Because the shear rate is higher near the solid wall, the impact of the viscosity parameter, which can be considered as the same of fluid viscosity, on temperature profiles is more obvious. It takes longer for fluid molecules to transfer energy to neighbouring molecules. Therefore, the temperature curves demonstrate a increasing nature/behaviour.

**Figure 12:**
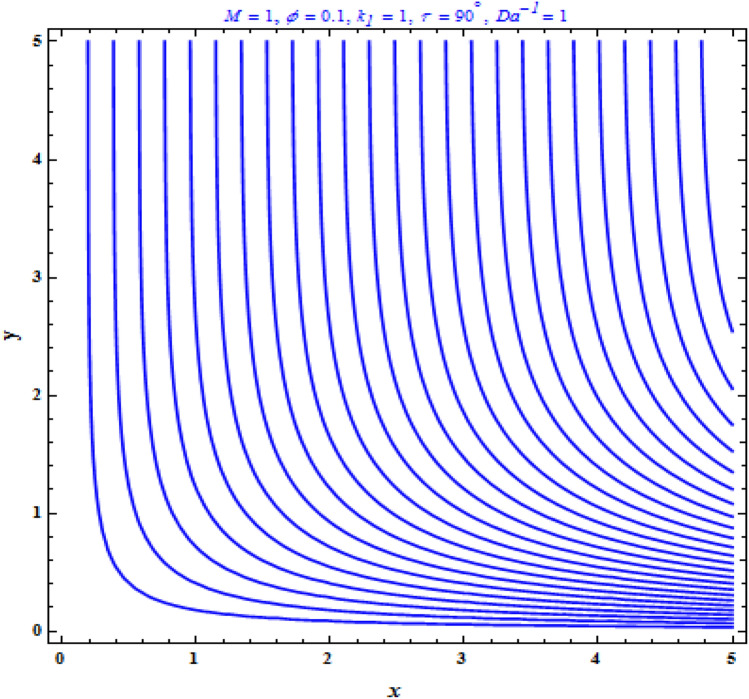
Streamlines for stretching boundary.

Figure 12 illustrates the flow patterns for different parameters. In this section, we’ll look at how to simplify techniques in the streamline circumstances of stretching cases. Highlights the pattern of streamlines for stretching boundary for various values of magnetic parameter with fixed parameters $k_{1} = Da^{ - 1} = 1,\,\phi = 0.1$. The flow field is regularized when the magnetic field occurs at particular subsequent places, as predicted by the physical theory. On the other hand, results in the removal of the streamline in the circular configuration.

## Conclusions

The Cattaneo- Christov heat flux is studied in carbon nanotubes with porosity in boundary layer flows of two viscoelastic fluids across a stretching surface. In many other terms, the parameters as with inclined magnetic field, viscoelastic constraint and Prandtl number strictly reduce the relaxation time coefficient. The analytical explanation of heat transfer presences in term of the Appell hypergeometric function. The temperature distribution inside the stretching/shrinking sheet is also controlled by the Prandtl number, thermal radiation, mass transpiration, heat source/sink, and magnetic parameter variables.

- Axial and transverse velocities increase as the parameters such as magnetic parameter, Darcy number increase in both SWCNTS and MWCNTs cases.
- The effect of the thermal radiation parameter raises the temperature enhances it.
- When the value of the viscoelastic parameter is enhanced, the fluid temperature increases in both stretching/shrinking conditions.
- The results of this research are very similar to those of Jafarimoghaddam et al. ^40^ in the absence of a magnetic parameter and when $Da^{ - 1} \, = \,N_{R} = 0$.
- The Mahabaleshwar et al. (2014) flow is recovered from Eq. (8) for $M = 1\,,\,\,k_{1} = \,1,\,\,Da^{ - 1} \, = \,0$.

## List of symbols

*B*_0_: Magnetic field [Tesla]
*c*: Constant rate of stretching [s^-1^]
*k*^***^: Mean absorption [−]
*K*: Permeability [m^2^]
*M*: Hartmann number [–]
*N*_*R*_: Thermal radiation parameter [–]
$$Pr$$: Prandtl number [–]
$q_{r}$: Radiative heat flux [W m^-2^]
*T*: Temperature [K]
$T_{\infty }$: Temperature far away from the sheet [K]
$T_{w}$: Temperature at the wall [K]
*u, v*: Velocities [m s^-1^]

## Greek symbols

$$\alpha$$: Thermal diffusivity [*m*^*2*^* s*^*-1*^]
γ: Relaxation time parameter [−]
σ_*f*_: Electrical conductivity [−]
σ^*^: Stefan-Boltzmann constant [W m^-2^ K^-4^]
μ: Dynamic viscosity [kg m^-1^ s^-1^]
ν: Kinematic viscosity [m^2^ s^-1^]
κ: Thermal conductivity [W m^-1^ K^-1^]
ρ: Density [kg m^-3^]
η: Similarity variable [−]

## Abbreviations

CNTs: Carbon nanotubes [−]
*f*: Base fluid [−]
MWCNTs: Multi-walled carbon nanotubes [−]
*nf*: Nanofluid [−]
ODEs: Ordinary differential equations [−]
PDEs: Partial differential equations [−]
SWCNTs: Single-walled carbon nanotubes [−]
*w*: Wall [−]
